# Optimized 3D Culture of Hepatic Cells for Liver Organoid Metabolic Assays

**DOI:** 10.3390/cells10123280

**Published:** 2021-11-24

**Authors:** Christian Moya Gamboa, Yujue Wang, Huiting Xu, Katarzyna Kalemba, Fredric E. Wondisford, Hatem E. Sabaawy

**Affiliations:** 1 Rutgers Cancer Institute of New Jersey, Rutgers University, New Brunswick, NJ 08901, USA; Christian.9107@gmail.com; 2 Department of Medicine, Robert Wood Johnson Medical School, Rutgers University, New Brunswick, NJ 08901, USA; yw429@rwjms.rutgers.edu (Y.W.); hx89@gsbs.rutgers.edu (H.X.); katarzynamkalemba@gmail.com (K.K.); 3 Department of Pathology and Laboratory Medicine, RBHS-Robert Wood Johnson Medical School, Rutgers University, New Brunswick, NJ 08901, USA

**Keywords:** liver regeneration, 3D culture, organoids, HepG2 cells, gluconeogenesis, metabolic assays

## Abstract

The liver is among the principal organs for glucose homeostasis and metabolism. Studies of liver metabolism are limited by the inability to expand primary hepatocytes in vitro while maintaining their metabolic functions. Human hepatic three-dimensional (3D) organoids have been established using defined factors, yet hepatic organoids from adult donors showed impaired expansion. We examined conditions to facilitate the expansion of adult donor-derived hepatic organoids (HepAOs) and HepG2 cells in organoid cultures (HepGOs) using combinations of growth factors and small molecules. The expansion dynamics, gluconeogenic and HNF4α expression, and albumin secretion are assessed. The conditions tested allow the generation of HepAOs and HepGOs in 3D cultures. Nevertheless, gluconeogenic gene expression varies greatly between conditions. The organoid expansion rates are limited when including the TGFβ inhibitor A8301, while are relatively higher with Forskolin (FSK) and Oncostatin M (OSM). Notably, expanded HepGOs grown in the optimized condition maintain detectable gluconeogenic expression in a spatiotemporal distribution at 8 weeks. We present optimized conditions by limiting A8301 and incorporating FSK and OSM to allow the expansion of HepAOs from adult donors and HepGOs with gluconeogenic competence. These models increase the repertoire of human hepatic cellular tools available for use in liver metabolic assays.

## 1. Introduction

Liver diseases account for about two million deaths annually worldwide [1]. Chronic liver diseases are caused mainly by viral infections, excess alcohol intake, fatty liver, autoimmune, and drug-related liver diseases and hepatocellular carcinoma (HCC) [2]. Non-alcoholic fatty liver disease (NAFLD) comprises fatty liver (steatosis), non-alcoholic steatohepatitis (NASH), and fibrosis/cirrhosis and may also lead to HCC. NAFLD is tightly associated with common metabolic disorders, such as obesity, metabolic syndrome, and type 2 diabetes mellitus (T2DM) [3]. Metabolic and physiological shifts in many liver diseases are linked to the gluconeogenic pathway [4,5,6,7]. For instance, insulin resistance initiates liver steatosis by providing substrates for mitochondrial β-oxidation such as glucose and glycerol [8]. This increased gluconeogenesis and elevated liver glucose production contribute to fasting hyperglycemia in T2DM patients [8]. Overall, studies of liver metabolism are limited by the inability to model and expand primary human liver cells in culture. Moreover, the effects of hepatic cell culture conditions on glucose production and gluconeogenic functions have not been well-characterized.

The liver architecture forming the hepatic lobule is primarily composed of two types of epithelial parenchymal cells: the hepatocytes and biliary epithelial cells (also known as cholangiocytes), while non-epithelial stromal cell types include macrophages (Kupffer cells), stellate cells, and sinusoidal endothelial cells [9]. The cell-of-origin of the hepatocyte-driven liver repair remains undetermined. Recent studies showed that non-parenchymal cells might play essential roles in hepatocyte repopulation following liver injury [10,11,12,13,14]. It is thought that the cholangiocyte-like oval cells may represent activated liver stem cells with bi-lineage potential, capable of regenerating cholangiocytes as well as hepatocytes [15,16]. Therefore, different cell types constitute the starting material for primary hepatic cell cultures.

Three-dimensional (3D) multicellular spherical culture models have consistently shown superiority in reproducing the in vivo behavior and drug resistance over standard two-dimensional (2D) monolayer cultures [17,18,19,20,21]. The 3D culture models can be largely classified into four types: spheroids; grown in scaffold-free conditions, spheres; obtained by expansion of adult stem-like cells (ASC) in growth factor supplemented serum-free conditions, organotypic slices; obtained by slicing of primary tissues, and organoids. Spheroid cells when introduced into scaffold-based natural or synthetic extracellular matrix (ECM) [22] showed enhanced functionality and intercellular interactions [23]. While the term “sphere” has been used to represent scaffold-based 3D culture, and organoids (at least cerebral organoids) have been generated in scaffold-free conditions, for clarity, we limit the use of the term ‘sphere’ or ‘spheroid’ to ‘scaffold-free’ 3D cultures, while we reserve the term ‘organoid’ to broadly represent 3D multicellular ‘scaffold-based’ cultures in ECM, derived from single stem-like or progenitor organoid forming cells. Accordingly, organoids are self-organizing scaffold-based units that are grown in serum-free conditions and display the full spectrum of cellular types in a tissue. Organoids have been obtained from ESC, iPSC, ASC, or stem-like tumor and progenitor cells [24,25,26].

Multiple protocols have been developed for generating 3D liver organoids [11,27,28,29,30,31,32,33,34]. Hepatoma cells could also be expanded in 3D cultures using the hanging drop method and/or with ECM hydrogels for drug metabolic assays [35,36]. Previous studies reported variations of serum-free 3D culture conditions for generating liver and hepatoma organoids [29,30,31,33,34,37]. Growth factor supplementation in these culture conditions was guided by the knowledge of the molecular pathways regulating liver embryonic development. These include the FGF, HGF, Wnt, BMP, RA, and TGFβ pathways that promote hepatic progenitor maturation, migration, and survival [38]. Clevers and colleagues reported the successful isolation, expansion, and differentiation of liver cells into liver organoids [31,34]. These initial hepatic organoid culture conditions were based on modifying their original intestinal organoid methods [39], which included epithelial cell culture within a 3D laminin-and collagen IV-rich ECM; Matrigel, together with supplementation with EGF (a mitogen used in seminal hepatic organoid studies [40]), Noggin (a BMP inhibitor), and R-spondin-1 (Rspond1) (an enhancer of WNT signaling and ligand of the stem cell protein LGR5) [39]. These studies suggested that adult liver cells from humans, but not mice, require regulation of TGFβ signaling and cAMP activity for long-term expansion [31,34]. TGFβ induces epithelial-to-mesenchymal transition (EMT) or epithelial plasticity through BMPs, depending on the developmental context [41]. Specific inhibition of TGFβ type I receptors (Alk4/5/7) by the small molecule inhibitor A8301 (albeit at different concentrations in prior hepatic organoid cultures [31,34,42,43]) extended the time and colony-forming efficiency before cultures eventually deteriorated. Further improvements in the generation of hepatic organoids from liver cells could be achieved by the addition of a GSK-β inhibitor (CHIR99021) and FGF7 and removing FSK from the culture media [33]. Still, postnatal hepatic organoids displayed higher expansion capacity than adult organoids [33,44]. Therefore, adult donor-derived human organoids may have a limited and/or impaired expansion or additional factors supporting hepatocyte maintenance and regeneration are necessary. We examined multiple organoid culture conditions and assessed the effects of oncostatin M (OSM); a member of the IL-6 family of cytokine linked to liver bud formation during embryogenesis [45,46], FSK; a diterpene highly efficient at increasing concentrations of cAMP [47], and the inhibitor A8301; causing downregulation of TGF-β, which correlated with the specification of hepatoblast towards the hepatocyte fate [48].

Here, we describe hepatic organoid 3D cultures using hepatoma and primary donor-derived human hepatocytes and report the effects of different media conditions on key genes associated with the gluconeogenic pathway (PCK1 and G6PC). We provide an optimized organoid culture condition in a single culture medium combination that limits A8301 and includes FSK and OSM to allow the expansion of HepG2 cells in HepG2 3D organoid cultures (HepGOs) with hepatocyte-like function and metabolic competence. This condition also allowed the expansion of primary donor-derived adult hepatic organoids (HepAOs) in 3D cultures.

## 2. Materials and Methods

### 2.1. The Materials and Human Liver Cells Used for the Generation of Hepatic Organoids

Primary human liver tissues from non-transplantable whole livers or resected healthy liver tissues were obtained from Lonza. Donors were healthy Caucasian males with no alcohol, tobacco, or drug abuse. Each tissue was procured in an ethical manner from consenting donors. Tissues were screened for infectious diseases and were negative for HIV, HBV, and HCV. Fresh human hepatocytes in suspension plates with viability > 85% were utilized. Under protocols approved by Lonza and Robert Wood Johnson Medical School Institutional Review Board (IRB) committees; the samples were immediately centrifuged at 200× *g* for 5 min at 8 °C. The supernatant was discarded and 15 mL of wash medium was added to resuspend the hepatocyte cell pellet. The centrifugation procedure was repeated twice with wash medium and once with basal medium. The pellet was resuspended using 15 mL of basal medium and the cells were counted using a hemocytometer. Primary human hepatocyte suspensions in multi-well plates were either freshly frozen or utilized in experiments.

### 2.2. Single Cell Derived Human Primary Hepatic Organoid Culture

Human hepatic cells were counted and 3 × 10^3^ cells were grown in our defined isolation medium (Supplementary Tables S1 and S2) and seeded at a concentration of 100 cells per microliter of the ECM matrigel (Corning, Corning, NY, USA). The single cell-ECM suspension mix was seeded in 24 well plates (Greiner Bio-One, Monroe, NC, USA) that had been incubated for 24 h prior to use at 37 °C. A total of 50 μL of Matrigel were added to the center of each well to form a dome-shaped chamber and incubated for 5–8 min at 37 °C before adding 500 μL of isolation medium to each well (Supplementary Table S2). Single cells were marked at the bottom of the plate and monitored for single-cell gradual expansion to form hollow spheres. After single cell-derived organoid formation, organoid growth was monitored every other day by recording bright phase-contrast images. Half of the media in each well were replenished every two days by carefully tilting the plate to a 45° angle to maintain plate organization, which was confirmed by matching the images to plate labeling after media exchange. Hepatic organoid growth was monitored during the first week of culture and single cell-derived organoids comprising each of at least 50–60 cells or more and forming at least a 100 μM organoid or larger were counted and recorded from the matching images under different culture conditions.

### 2.3. Single Cell Derived Human Primary Hepatic Organoid Expansion

After at least 4 days, the isolation media was substituted for EM containing 10 μM ROCK inhibitor (Y-27632) (Supplementary Table S2). The EM was replenished three times per week. After the first 6 days in culture, the ROCK inhibitor was removed from the media. The cells within the ECM mix were expanded for 14 days to form organoids. In the initial optimization studies for determining optimum components expansion media (EM), we utilized a variety of small molecules and growth factors, including Wnt agonists such as R-spondin1 and CHIR99021; EGF, FGF7, and FGF10; HGF and the TGFβ inhibitor A8301, at different combinations (conc.), about one log conc. above and below the previously reported conc., guided by prior reports. For example, A8301 has been reported to have been used at 50 nM [42], 5 μM [31], 2 μM [33], and 4 μM [43] for generating hepatic organoids. We assessed this range of A8301 conc., and the media conditions were tested for the ability to support growth. During the first week of 3D culture, small organoids emerged from Matrigel-embedded hepatocytes in select conditions. We selected those conditions that also resulted in 90 ± 5% cell viability at day 7 for further examination. This allowed us to refine these conditions, and eventually determine the EM conditions that resulted in robust organoid expansion. After 14 days, the media was supplemented with 25 ng/mL of BMP7, and the organoids were cultured for 5 days before replacing the media with hepatocyte differentiation medium (DM) (Supplementary Table S2). The DM were replenished every 3 days. On day 7 of differentiation, the organoids were used for analyses.

### 2.4. Single Cell Derived Hepatic Organoid Proliferation Rates

For generating the cell survival and proliferation rates of HepAOs and HepGOs, the organoids grown in expansion medium were dissociated into single cells with Accutase. Cell numbers were counted by trypan blue exclusion at the indicated time points. Based on the basic formula of the expansion curve y(t) = y0 × e^(growth rate×t)^ (y = cell numbers at final time point; y0 = cell numbers at initial time point; t = time) and previous reports about human primary hepatic organoids proliferation rates [31], we derived the proliferation rate of HepAOs and HepGOs. The doubling time was calculated based on the basic formula of doubling time = ln(2)/growth rate for each time window that was analyzed.

### 2.5. Generation and Harvesting of Wnt-3a Conditioned Media

Wnt-3α-expressing cells were purchased from ATCC (CRL-2647). Cells were cultured in Greiner Bio-One CELLCOAT™ tissue culture-treated T-75 flasks at an initial concentration of 1.5 × 10^6^ cells. The cells were cultured in 20 mL of Wnt-3α growth medium (WGM) (Supplementary Table S1) and passaged once 75% confluency was reached. We used 0.4 mg/mL G-418 for selection and cells were kept growing on selection medium for a week before producing conditioned medium. The cells were lifted using 3 mL of TrypLE (Life Technologies, Carlsbad, CA, USA) and passaged. The process was repeated until approximately 40–50 T75 flasks were generated. The media were replaced with 100 mL of harvest medium (HM) (Supplementary Table S1). The cells were then incubated in HM for one week without media changes. After one week, the media were collected, and tubes were centrifuged at 500× *g* for 5 min at 8 °C. Following this step, the media were filtered using filter cups (Stericup Quick Release, Millipore, Burlington, MA, USA). Lastly, 25 mL of the collected harvested medium was separated into 50 mL canonical tubes and utilized fresh or stored for no more than 2 weeks at 4 °C. Recombinant human Wnt-3α (R&D, 5036-WN010) was used at a final conc. of 100 ng/mL as a positive control, and Wnt activity of conditioned medium could be examined by the TOP/FOP assay as described [49].

### 2.6. Plating and Generation of R-Spondin-1 Conditioned Media

R-Spondin-1-expressing 293T cells were purchased (EMD Millipore, SCC111) and cultured in Greiner Bio-One CELLCOAT™ tissue culture-treated T-75 Flasks at an initial concentration of 1.5 × 10^6^ cells. The cells were cultured in 20 mL of R-Spondin-1 Growth Medium (RGM) (Supplementary Table S1) and passaged once 75% confluency was reached using TrypLE (Life Technologies). The media was changed with fresh RGM every 2 days. To harvest the R-Spondin-1 conditioned media, once cells reached 70% confluency, the media was replaced with 100 mL of HM (Supplementary Table S1). The cells were then incubated in this medium for one week without media changes. After this week, the media was collected in 50 mL canonical centrifuge tubes. The tubes were centrifuged at 500× *g* for 5 min at 8 °C. Following this step, the media were filtered using 500 mL filter cups (Millipore), and harvested media were aliquoted into 15 mL canonical tubes and utilized fresh within two weeks or stored at −20 °C.

### 2.7. Hepatoma Cell Organoid Culture

Well-differentiated hepatoma cell lines HepG2, PLC/PRF/5, and HuH7 were maintained as previously described [21]. In initial studies, we used classical culture media containing high glucose concentration (4.5 g/L) corresponding to systemic hyperglycemia. Low glucose concentrations corresponding to normoglycemia (1 g/L) were utilized in parallel to study the effects on single cell-derived cell growth. HepG2 cells were first cultured in a monolayer. Once 80–90% confluence was reached the cells were collected and used to start the single cell-derived organoid culture. HepGOs were generated on 48 well tissue culture plates (Greiner Bio-One, Monroe, NC, USA). The plates were left overnight at 37 °C before using them to plate the Matrigel matrix. Cells were plated at a concentration of approximately 40 cells per μL of Matrigel (Matrigel final conc. was 80%). We used a 25 μL drop/well to form the organoid dome at the center and the organoid dome was overlaid with 250 μL of human expansion medium (EM) (Supplementary Table S2) with the multiple EM variations tested. The plates were then incubated for 7 days under standard tissue culture conditions (37 °C and 5% CO2). Media was changed every other day. HepGO growth was monitored during the first week of culture and single cell-derived HepGOs comprising each of at least 50–60 cells or more and forming at least a 100 μM or larger organoid were counted and recorded from the matching images under different culture conditions. After at least 7 days of expansion, the media was replaced with differentiation media (DM). The organoids were cultured for an additional 7 days, at which point the organoids were used for analysis.

### 2.8. Recovering of Organoids from 3D Culture

Recovery of organoids was performed as described for other organoid types [50,51] with modifications. In order to use the organoids for analyses, it is necessary to remove them from the Matrigel. To accomplish this, hepatocyte DM were removed from the wells using a 200 μL pipette. The plate was placed on ice, then by using an unfiltered 1000 μL cold pipette tip, 500 μL of ice-cold PBS buffer was transferred into each well. The PBS was allowed to remain in the wells for 3–5 min before collecting it into ice-cold 15 mL tubes (placed on ice 30 min before starting the procedure). The tubes containing the organoids were then centrifuged at 1200 rpm for 4 min at 4 °C. The supernatant was removed leaving the organoid pellet intact. Next, 500 μL of cold cell recovery solution was added to the pellet. The pellet was dissolved in the solution by gentle pipetting up and down slowly multiple times. The solutions were then transferred to 1.5 mL Eppendorf tubes and left on ice for one hour. The tubes were gently vortexed every 20 min. After one hour, the tubes were centrifuged at 4000 rpm for 5 min, and the supernatants were discarded. The pellets were washed twice using 1 mL of ice-cold PBS with centrifugation and recovered organoid cells were used for different assays.

### 2.9. Assessment of Expression of Hepatic and Gluconeogenic Targets in Organoids

RNA was first extracted from the differentiated organoids using Trizol. RNA was used for cDNA synthesis using the iScript Kit (BioRad, Hercules, CA, USA). Quantitative PCR (Q-PCR) was performed for targets of interest including G6PC, PCK1, and AFP using Bio-Rad CFX96. Bio Rad’s SYBR Green supermix was used with the target-specific primers (Supplementary Table S3). The data were analyzed using Excel and Prism 8.

### 2.10. Immunofluorescence Staining and Imaging of Hepatic Organoids

IF and sample processing were performed generally as previously described [52,53]. Prior to being fixed, the organoids were recovered from the Matrigel After washing three times with PBS. The organoids were then fixed using 4% PFA and incubated at 37 °C for 15 min. The organoids were allowed to precipitate to the bottom of the plate before carefully removing the fixative. The samples were washed three times with PBS and stored at 4 °C before the permeabilization step was done. For permeabilization, the PBS was removed and replaced with 500 μL of PBS-0.5% Triton X-100. The organoids were then incubated in the PBS-Triton X-100 solution overnight at 4 °C. The following day, the solution was removed and the organoids were washed three times with PBS. The treated organoids were used immediately for immunostaining for human-specific albumin and HNF4α hepatocytic markers. For all blocking and antibody dilutions, a 5% BSA solution was used as specified by the manufacturer’s instructions. Omission of the primary antibody resulted in no background staining. DAPI was used to stain nuclei at a 1:200 dilution, albumin and HNF4α were utilized at 1:200 and 1:100 dilution, respectively (Supplementary Table S4). Whole-mount staining was performed and the organoids were imaged using a Nikon A1R Si Confocal microscope.

### 2.11. Confocal Microscopy-Based Volumetric Measurements of Organoids

Confocal scanning microscopy-based volume measurements were conducted by demarcating cytosolic ALBUMIN and nuclear DAPI. Samples were scanned at multiple vertical z-steps to determine the distance between the bottom and the top of the cell at every xy position using water-immersion objectives to minimize the axial mismatch Model [54]. Volume calculation on segmented images was done using ImageJ plugins Volumest (http://lepo.it.da.ut.ee/~markkom/volumest/ (accessed on 29 February 2020)) and Volume Calculator (http://imagej.net/Volume_Calculator (accessed on 29 February 2020)).

### 2.12. Glucose Production Assays

Prior to each glucose production assay, organoid cells were serum starved for 3 and 24 h. The assay was done in 3D organoid culture media, containing no glucose or pyruvate. For substrate glucose production assays, the glucose production basal media (BM) were supplemented with either glycerol, glucagon, or a combination of both. Data were obtained by obtaining media from wells that contained 12–14 Matrigel droplets containing approximately 1400 organoids. Glucose measurements were done enzymatically with Glucose Assay Kit (Abcam, Boston, MA, USA).

### 2.13. Statistical Analysis

Statistical analyses were performed using GraphPad Prism 8 (GraphPad Software Inc. (San Diego, CA, USA), www.graphpad.com (accessed on 6 August 2019)). Data are presented as the mean ± standard deviation (SD). Statistical significance was determined by *t*-test and ANOVA, with Dunnett’s post-test when appropriate. A *p*-value < 0.05 is represented by a single asterisk, a *p*-value < 0.01 is represented by a double asterisk, three asterisks indicate *p*-value < 0.001, while four asterisks indicate *p*-value < 0.0001.

## 3. Results

We first considered multiple well-differentiated hepatoma cell lines (HepG2, PLC/PRF/5, and HuH7) in 3D culture for their abilities to form serum-free and BSA-free hepatic 3D cultures. Among these cells, we analyzed the effect of glucose on cell growth. HepG2 cells could proliferate faster and their 3D culture size is smaller than that of HuH7 and PLC/PRF/5 cells. HuH7 and PLC/PRF/5 cells could grow in 2D culture but HuH7 became elongated and slowly stopped dividing, while PLC/PRF/5 cells formed smaller and fewer cultures than HepG2 cells under 3D conditions. When cultured under low glucose concentrations corresponding to normoglycemia (1 g/L), the cell growth of HepG2 cells was not significantly affected, while the growth rate of HuH7 cells was highly reduced in normoglycemic conditions, similar to previous reports [55], suggesting that HepG2 cells could be utilized to optimize the hepatocyte-like and metabolic functions in 3D organoid cultures.

### 3.1. Expansion Rates and Morphological Changes in 3D Culture Conditions

**Figure 1:**
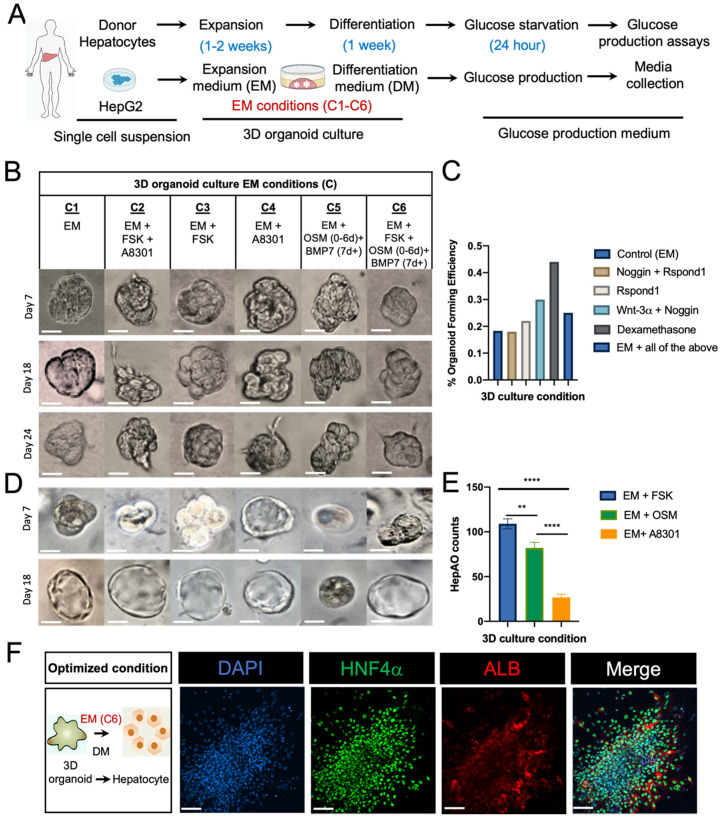
Human hepatic organoids in 3D culture. (**A**) Schematic of the single cell derived hepatic organoid generation process. Adult donor-derived hepatocytes and/or HepG2 cells were subjected to 3D culture, expansion in expansion media (EM) under various supplemental conditions, and differentiation in hepatocyte differentiation media (DM), followed by molecular assays for hepatocyte-specific markers and glucose production assays for gluconeogenesis. (**B**) Top, various EM conditions (C1–C6) tested on primary hepatocytes and HepG2 cells. Brightfield images of human-derived organoids derived from dissociated single cells after expansion for the indicated times in EM supplemented with growth factors and small molecules (FSK, Forskolin, OSM, Oncostatin M, A8301). Representative images from conditions that allowed the formation of organoids at day 7 with cell viability at 90 ± 5%. (**C**) Percentage organoid forming efficiency from primary hepatocytes in 3D organoid cultures (HepAOs) in different media conditions. (**D**) Bright field images of expanded primary hepatocytes in 3D organoid cultures (HepAOs) grown in different media conditions. Experiments were with hepatocytes derived from three different donors. (**E**) Number of HepAOs generated using three distinct small molecules EM conditions. (**F**) Hepatic organoids generated in C6 EM expanded for 4 weeks, followed by Differentiation Media (DM) with BMP7 for 2 weeks. Organoids were passaged twice followed by IF. Respective stains: DAPI for nuclei, HNF4α for hepatocytes, ALBUMIN (ALB) for hepatocyte function. All experiments were performed in triplicate unless otherwise stated. Scale bar is 100 μm. Comparison of counts and conditions were determined by two-way ANOVA with Bonferroni post hoc test (**** *p* < 0.0001, ** *p* < 0.01).

The Different sets of EM conditions were tested for their potential to generate HepG2 modified hepatic-like organoids (HepGOs). In parallel, freshly isolated human cells were grown in isolation media in order to select and enrich the ASC fraction harboring the hepatic organoid forming cells [31,33]. We initially tested various EM conditions for the ability to support growth. During the first week of 3D culture, small organoids emerged from Matrigel-embedded hepatocytes in select conditions. We selected the conditions that demonstrated >90 ± 5% cell viability at day 7 for further examination. These experiments allowed us to refine the initial culture conditions, and eventually determine the final optimized conditions that resulted in robust organoid growth. The optimization studies were particularly critical for components such as the TGFβ inhibitor A8301, which was utilized at a conc. range from 50 nM to 4 μM in prior hepatic organoid studies [31,34,42,43]. A8301 conc. from 5–10 μM resulted in culture deterioration at day 7 and were excluded. We included A8301 at 0.5 μM, which is a conc. within the lower range of that previously reported, in our tested conditions (Figure 1A,B). Single-cell suspensions were utilized in 3D single cell-derived organoid culture in various optimized conditions (Figure 1A), which were tested for the ability to support the growth and the expansion rates of organoids in EM alone (Condition C1) or supplemented with different growth factors/small molecules (Conditions C2–C6) (Figure 1A–E and Supplementary Table S2).

During the first week of culture, small organoids emerged from Matrigel-embedded hepatocytes in all culture conditions (Supplementary Figure S1A,B). Hepatic organoids demonstrated a typical grape-like appearance at day 7 (Figure 1B). The organoids expanded from a diameter of 100 μm in one week to a diameter of 400–500 μm within 4 weeks and could be passaged by mechanical dissociation at a ratio of 1:3 every 7–10 days. These collective studies allowed us to refine conditions, eventually resulting in robust organoid growth (Supplementary Figure S1A,B). In 3D organoid cultures from both HepG2 cells (HepGOs) (Figure 1B and Figure S1A) and adult donor-derived primary hepatocytes (HepAOs) (Figure 1 and Figure S1B), morphological evolution was observed throughout the organoid development process. Different molecules appeared to facilitate the expansion of projections in the HepGOs around day 24 (Figure 1B) and the repopulation of the lumen in the differentiated HepAOs (Figure 1D and Figure S1B). It has been previously shown, with the exception of OSM, that the supplemented growth factors/small molecules can alter the expansion rates of organoids in 3D cultures [31,34]. We assessed the organoid forming efficiency at one week in single cell-derived organoids (Figure 1B,C). There was no significant difference in the organoid forming efficiency when changing the core EM components, with a trend towards an increase in organoid forming efficiency with the addition of dexamethasone, an effect that was reversed when all core media components were included (Figure 1C). When examining 3D cultured derived from single hepatocyte cell suspensions from adult donors, while previously reported conditions (C1–C3) [31,34] resulted in similar expansion rates, conditions including F and OSM supplemented media (C5–C6) expanded rapidly over a period of 2 weeks, while conditions including A8301 supplemented media (C4) showed the least number of organoids being formed over the same period of time (Figure 1E).

Since serum-free and BSA-free EM condition C6 (EM + FSK + OSM) resulted in 3D organoid culture with an intricate morphology for both HepGOs and HepAOs upon long-term expansion (Supplementary Figure S1B), these hepatic organoids were cultured in the improved medium, with a consistent proliferation rate and doubling time of about 62 h (Supplementary Figure S1C). We examined and confirmed the expression of hepatocyte-specific HNF4α and ALB secretion upon induction of differentiation in these 3D organoids using Immunofluorescence (IF) assays (Figure 1F and Figure S1D).

### 3.2. Expansion of 3D Organoid Culture with EM and DM in HepGOs

**Figure 2:**
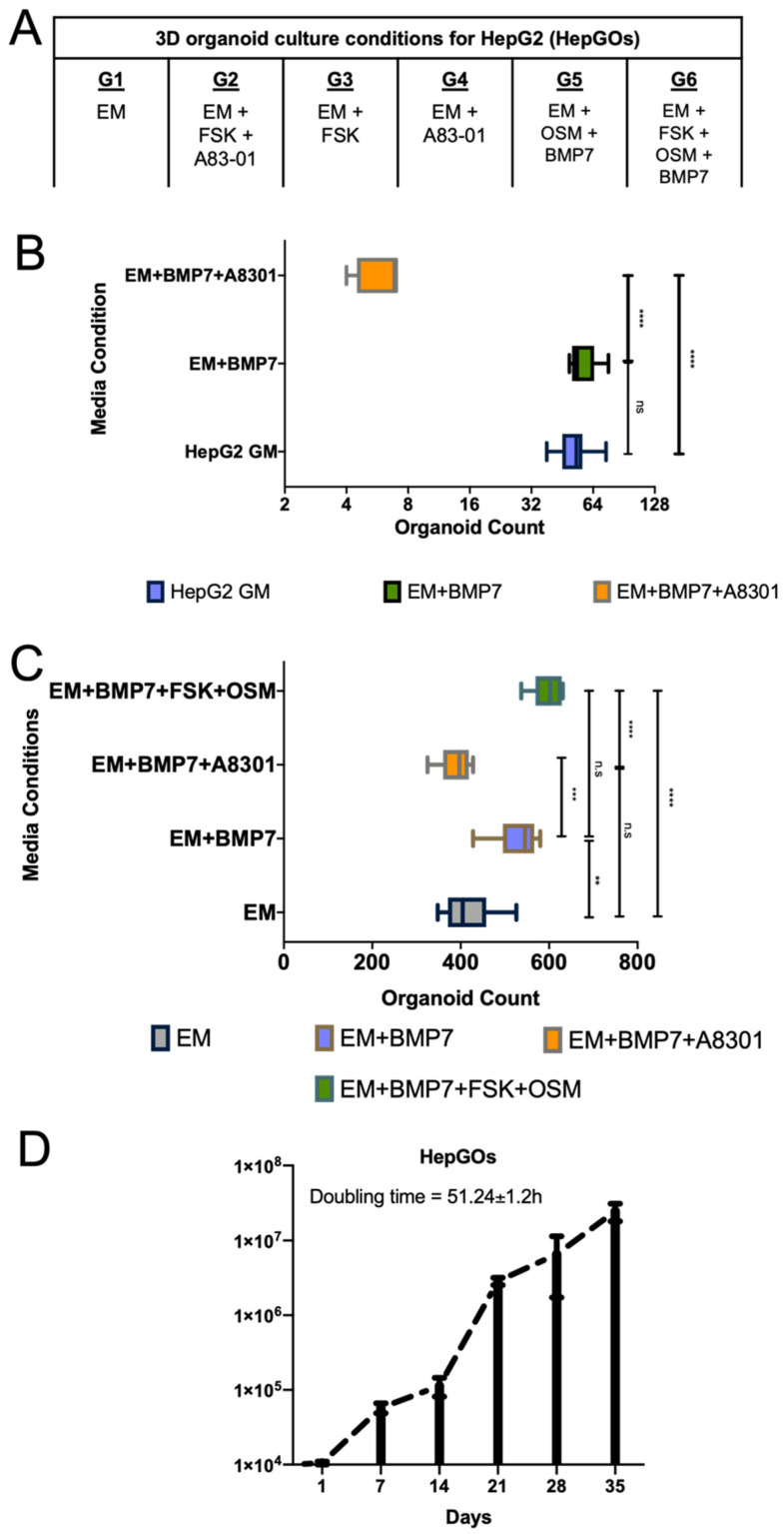
Expansion of HepG2 cells in 3D organoid cultures under different conditions. (**A**) Various EM with supplemental conditions, combined with differentiation in DM, for 3D culture of HepG2 cells to form HepGOs. (**B**) Counts of HepGOs derived using BMP7 and A8301 supplemented conditions, compared to regular HepG2 growth media (GM). (**C**) Counts of HepGOs organoids derived using various supplemented conditions. As expected, the number of organoids grown in conjunction with OSM and FSK is the highest when compared to the other conditions. (**D**) Proliferation rate of single cell-derived HepGOs and in vitro growth rate at the indicated time in optimized EM. Doubling time is indicated on days 21–28. Comparison of counts and conditions were determined by two-way ANOVA with Bonferroni post hoc test (**** *p* < 0.0001, *** *p* < 0.001, ** *p* < 0.01, ns, not significant).

BMP7 was shown to accelerate hepatocyte maturation [56], and the addition of BMP7 to the EM prior to the start of hepatocyte differentiation accelerated the formation of hepatocytes from primary liver tissues [31,34]. Therefore, we assessed the effects of adding BMP7 to HepG2 organoid 3D culture conditions. BMP7 slightly but not significantly improved HepGO number when compared to growing HepG2 cells in 3D culture using serum-containing growth media (HepG2 GM) (Figure 2A,B). On the other hand, conditions including the TGFβ inhibitor A8301 significantly limited the 3D culture and expansion of HepG2 cells (Figure 2B). This could be in part due to the ability of A8301 to limit Wnt-3α/β-catenin-induced cellular growth, or the context-dependent coregulation between TGFβ and BMP signaling [41]. In the absence of A8301 (EM + BMP7), HepG2 cells proliferated in 3D organoid cultures at a rate comparable to that of serum-containing 3D culture growth media (HepG2 GM) (Figure 2A). Moreover, the EM condition C6 (EM + FSK + OSM + BMP7) also supported the higher expansion of HepGOs when compared to EM only or EM + BMP7 (Figure 2C). HepGOs were cultured in the improved medium, with a consistent proliferation rate and doubling time of about 52 h (Figure 2D).

### 3.3. Expression of Gluconeogenic G6PC and PCK1 in Organoids

**Figure 3:**
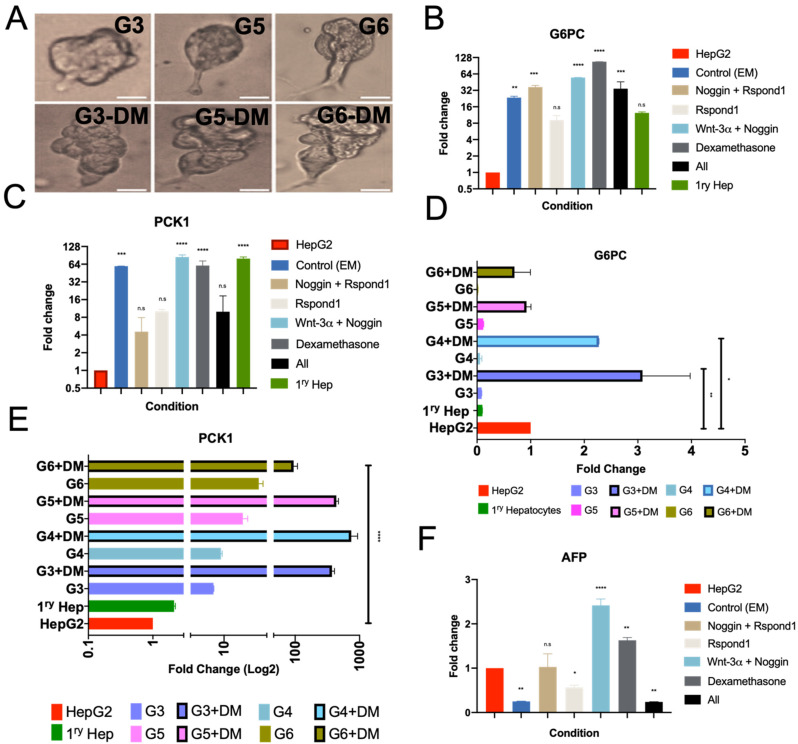
Gluconeogenic marker expression in 3D organoid cultures under different conditions. (**A**) Representative images of HepGOs derived under various conditions described in Figure 2A. HepGOs were expanded for one month, and then media were supplemented with 25 ng/mL of BMP7 for 5 days before replacing the media with hepatocyte DM for one week. Data are from experiments that were done in at least in duplicate with three replicates per condition and were normalized to the endogenous control (β-Actin) and HepG2 GM cultures. Scale bar is 100 μm. (**B**,**C**) Expression of G6PC and PCK1 in standard EM and EM supplemented with various core molecules. (**D**,**E**) Expression of G6PC and PCK1 in EM conditions (C3–C6) that produced the highest number of organoids. (**F**) AFP fold change of expression of HepGOs grown under different conditions. (**** *p* < 0.0001, *** *p* < 0.001, ** *p* < 0.01, * *p* < 0.05, ns, not significant).

Glucose-6-phosphatase (G6PC), and phosphoenolpyruvate carboxykinase-1 (PCK1) are key enzyme checkpoint regulators of gluconeogenesis, and differences in their expression levels have been linked to T2DM [4,5,57,58]. Compared to HepG2 GM alone, we first assessed the effects of the core EM components. The overall expression of G6PC and PCK1 were significantly increased (approximately between 8-to 64-fold) when HepGOs were cultured in 3D organoid EM (EM) compared to HepG2 GM (Figure 3A–C). For G6PC, EM supplemented with either a combination of Noggin and Rspon1, Wnt-3α and Noggin, dexamethasone, or all four (EM + Noggin, Rspon1, Wnt3α, and dexamethasone) resulted in a significant increase in the relative G6PC expression (Figure 3B). On the other hand, for PCK1, EM supplemented with either Wnt-3α and Noggin, or dexamethasone resulted in a significant increase in the relative PCK1 expression (Figure 3C). Interestingly, the addition of all of the tested factors and molecules caused a significant decrease in the expression of both G6PC and PCK1. Therefore, these data suggest that some of these factors may play either direct or indirect modulatory roles in the expression of G6PC and PCK1. Notably, EM supplemented with either Wnt-3α and Noggin, or dexamethasone resulted in a significant increase in expression of both G6PC and PCK1, with PCK1 reaching the level of primary hepatocytes (Figure 3B,C).

We next assessed the effects of the supplements to the EM forming the various EM C1–C6 conditions including variations of (EM + FSK + OSM + BMP7) on G6PC and PCK1 expression upon expansion for one month and prior to and after induction of hepatocyte differentiation (Figure 3A–C). The conditions (F + BMP7) and (A8301 + BMP7) resulted in induction of G6PC expression after differentiation (Figure 3D), while various conditions (EM + FSK + OSM + BMP7) induced PCK1 expression prior to differentiation and more after differentiation (Figure 3E). With our findings that EM conditions upregulate the expression of G6PC and PCK1 to nearly that of primary hepatocytes, we assessed the expression of HCC biomarker alpha fetal protein (AFP) [59]. HepGOs that were expanded in 3D EM supplemented with either Wnt-3α and Noggin showed a significant increase in the relative expression of AFP (Figure 3F). Intriguingly, HepGOs which were expanded in either EM alone, EM with Rspnd1, or with all four (EM + Noggin, Rspond1, Wnt3α, and dexamethasone) showed significant reductions in AFP expression (Figure 3F). Altogether, these findings suggest that EM core components and/or some of the supplemented molecules could be interacting with pathways linked to the transcription of AFP and hepatocyte maturation. While initially intriguing, understanding the effects of these molecules on AFP might facilitate the future development of new differentiation therapies for HCC.

### 3.4. Expansion and Freezing of 3D Cultured Hepatic Organoids

**Figure 4:**
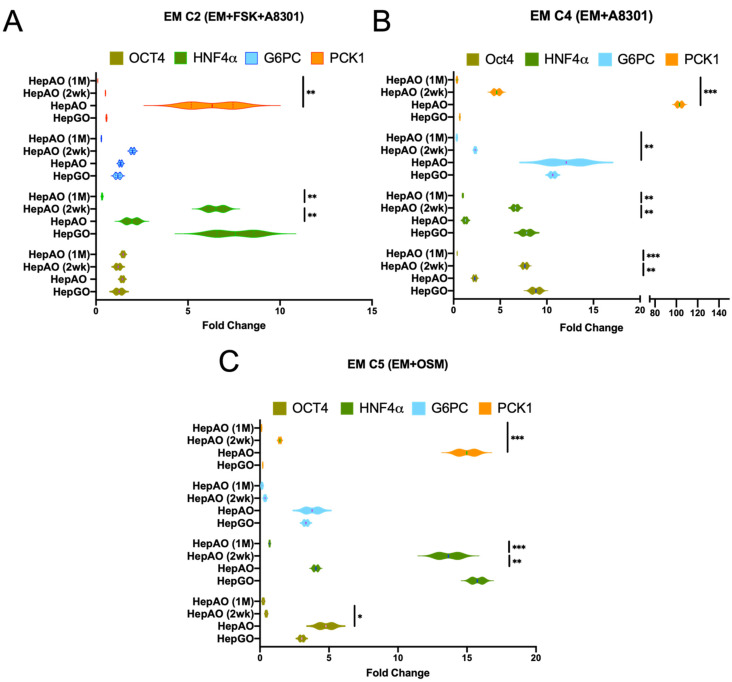
Expansion and freezing of human hepatic organoids in 3D culture. (**A**–**C**) Expression of OCT4 (surrogate marker for ASC), hepatic marker HNF4α, and gluconeogenic markers G6PC and PCK1 in HepGOs, HepAOs (immediately after EM), and HepAOs after 2-week (HepAO-2wk) or one month (HepAOs-1M) of organoid freezing and thawing under various EM conditions with FSK + A8301, A8301, and OSM (C3–C5). Data demonstrate the normalized fold change over base line expression in organoid cultures. Data are from experiments that were performed in at least in duplicate with (N = 3) donor hepatocytes and multiple HepG2 organoid cultures, each in three replicates per condition. (*** *p* < 0.001, ** *p* < 0.01, * *p* < 0.05).

We next assessed the effects of the tested key growth factors and small molecules in supplemented EM conditions on the expression of hepatocyte gluconeogenic target genes upon expansion of fresh or frozen HepAOs and HepGOs. Expression was assessed in HepG2 cells (HepG2), freshly isolated donor-derived human liver primary cell suspension (HPLC), two-week frozen and re-expanded HepAOs (HepAO-2wk), and one-month frozen and re-expanded HepAOs (HepAO-1M) (Figure 4).

Induction of PKC1 expression post DM (C1) was relatively similar among HepAOs from three donors (Supplementary Figure S2A), and organoids were enriched for OCT4 and SOX2 (Figure 4A–C and Figure S2B). The relative expression of hepatic gluconeogenic targets was steady and higher in fresh than in frozen and reconstituted organoids. When comparing various EM conditions, conditions with F countered those with A, as expected (Figure 4A,B), while OSM induction of G6PC and PCK1 expression tend to decrease at one-month post differentiation (Figure 4C).

### 3.5. Metabolic Competence of 3D Cultured HepGOs

**Figure 5:**
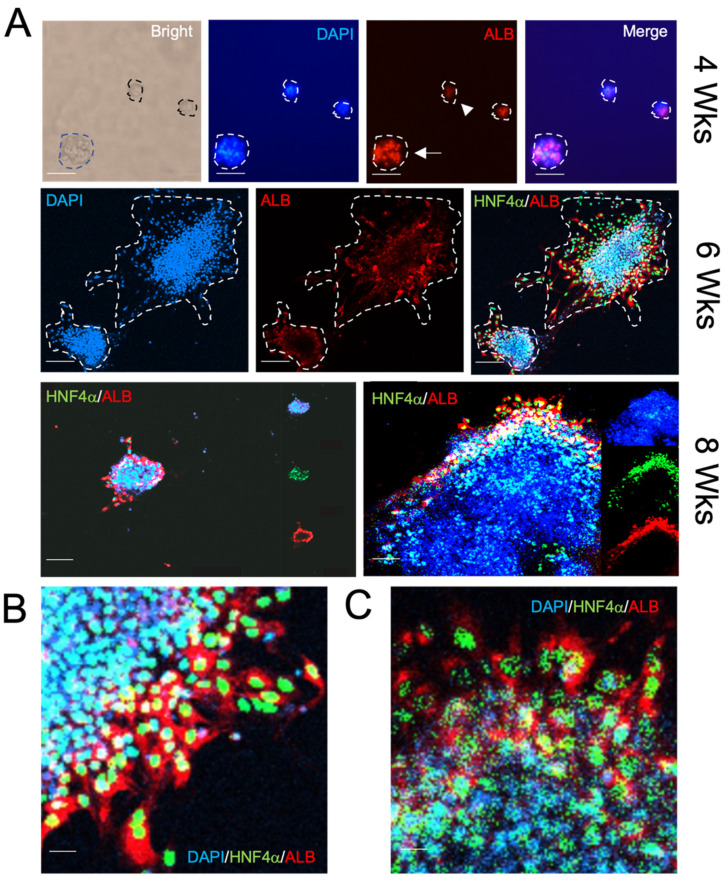
HepGOs show dynamic rates of expansion and expression of functional markers. (**A**) Representative IF images of HepGOs (white dotted line outlining each organoid) expanded for the indicated times. At 4 weeks, ALB staining in organoids (red) (arrow), while smaller cell structures (arrowhead) showed minimal or no staining. Note the expression of HNF4α (green) and the functional marker ALB (red) over DAPI, the nuclear marker in blue, becomes localized to the periphery of larger organoids (right side vs. left side images of organoids at 8 weeks). (**B**,**C**) Higher power IF images showing HNF4α and ALB-expressing cells within the peripheral area of HepGOs at 6 and 8 weeks, respectively. Scale bar is 100 μm in A and 10 μm in (**B**,**C**).

Maintaining the expression of functional hepatocytic markers over prolonged culture periods remains a challenge in both 2D and 3D organoid cultures. We performed IF assays to assess the presence of ALB and its continued expression in growing differentiated organoid cultures (Figure 5A).

**Figure 6:**
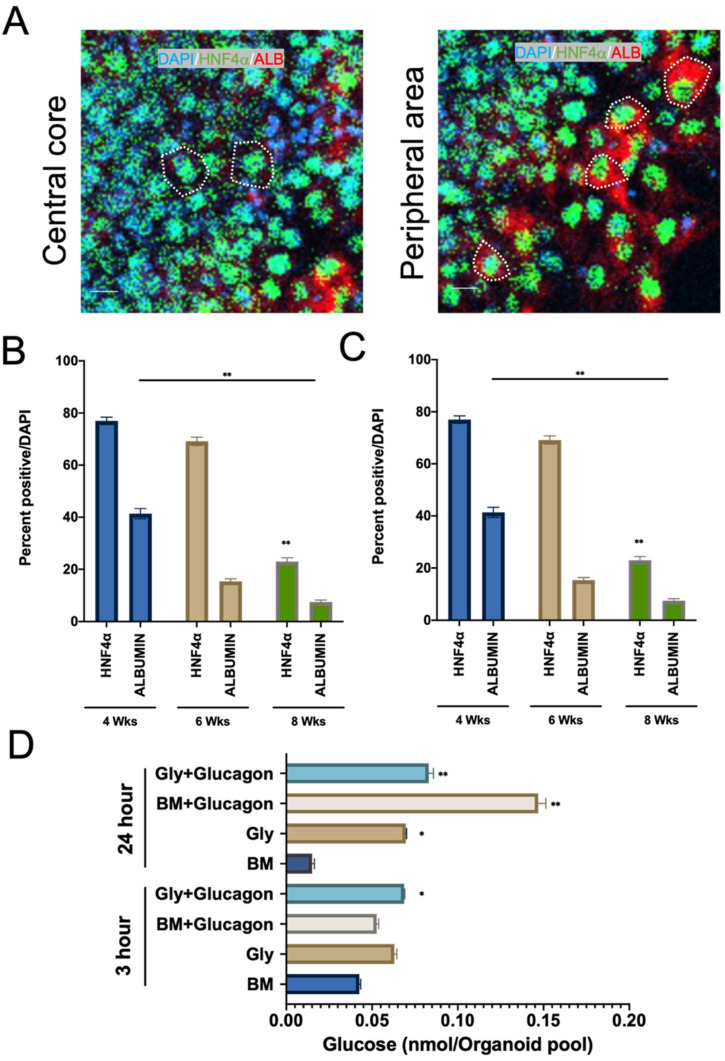
HepGOs show spatial distribution of HNF4α and ALB-expressing cells and metabolic competence. (**A**) High power IF images showing the central core and the peripheral area of HepGOs at 8 weeks. Representative cells within the central core and peripheral area of the same organoid are outlined with white dotted lines. The typical nuclear DAPI (blue) and HNF4α (green) overlay and cytosolic ALB (red) staining are outlined in the two zones. (**B**,**C**) Quantitation of expression of HNF4α (green) and the functional marker ALBUMIN (ALB) in HepGOs at the indicated times. (**D**) Glucose production in HepGOs expanded in C6 EM supplemented with F + OSM. The HepGOs were starved for 3 or 24 h and then allowed to produce glucose for 24 h. The glucose production basal media (BM) was supplemented with either glycerol, glucagon, or a combination of the two. Data were obtained from pooled organoids with a total of ~70,000 organoid cells. Scale bar is 10 μm. (** *p* < 0.01, * *p* < 0.05).

Upon expansion for one month and induction of differentiation for one week, HNF4α was significantly upregulated (Supplementary Figure S3A) and ALB could be detected for an additional 4 weeks (Figure 5B,C). Notably, at 8 weeks (4-weeks post EM and 3-weeks post induction of differentiation), HepGOs could be distinguished by volumetric analysis into two groups: small organoids (on average 181.43 mm deep × 92.50 mm wide) that showed the largest expression of HNF4α/ALB positive cells (Figure 5B,C and Figure S3B,C), while larger HepGOs showed a central expanding HNF4α/ALB negative core with more HNF4α/ALB-positive cells in the periphery (Figure 6A–C).

Next, we assessed metabolic functions related to gluconeogenesis in these 3D cultured organoids. Our group has recently reported the substrate preferences for gluconeogenesis in primary hepatocyte glucose production assays [60,61]. Moreover, we demonstrated the induction of the rate-limiting enzyme of gluconeogenesis, G6PC, upon starvation [61]. Since our 3D organoid culture conditions with EM supplemented with FSK + OSM generated HepGOs containing cells with hepatocyte-like function, we assessed glucose production upon growth factor starvation in these organoids to determine their metabolic competence. The organoids were serum starved for 3 and 24 h and allowed to produce glucose for 24 h. The glucose production media was supplemented with either glycerol, glucagon, or a combination of the two. Glucagon significantly enhanced glucose production in HepGOs after a 24 h starvation, while glycerol with glucagon had a similar effect in the 3 h glucose starvation assays (Figure 6D). These data suggest that HepGOs have metabolic features with cells capable of glucose production in 3D culture upon growth factor starvation.

## 4. Discussion

Hepatic organoids have offered an expanded platform to study human liver cells at the 3D level in scaffold-based serum-free defined conditions. Organoids have been derived from tissue-resident ASCs, iPSCs, and amniotic cells using different combinations of growth factors and small molecules [30,31,33,34,37,48,62,63,64,65,66]. These findings suggest that the generation of hepatic organoids can potentially be optimized for different studies. We have tested the capacity of different molecules to generate rapidly expanding and metabolically competent organoids that could express gluconeogenic target genes, which have been either sparingly shown or not discussed in previous studies [28,30,31,33,34,35,36,37,62,67]. Clevers and colleagues described the successful isolation, expansion, and differentiation of postnatal liver cells into hepatic organoids [31,34]. These studies demonstrated that up to one-third of mature EPCAM^+^ epithelial cells expand as cystic organoid structures when using normal adult liver tissues [31] or HCC cells [34]. They reported utilizing Matrigel, which acts as the ECM, with EM including the growth factors HGF, EGF, FGF, and Rspond1, TGFβ inhibitor A8301, and cAMP inducer FSK. Upon blocking Notch signaling and the addition of FGF19, BMP7, and dexamethasone for differentiation, human cells are fated toward a hepatocyte phenotype. These key studies suggested that adult liver tissue from humans, but not mice, requires the regulation of TGFβ signaling and cAMP activity for long-term expansion [31,34]. Further improvements in the generation of hepatic organoids from postnatal fetal cells could be accomplished by the addition of a GSK-β inhibitor (CHIR99021) and FGF7 and removing FSK from the 3D culture media [33]. Nusse and colleagues have reported similar culture conditions, with the exception of excluding Rspond1 and including the injury-induced inflammatory cytokine TNFα for an enhanced expansion of mouse hepatocytes [68]. These studies revealed that mouse and human fetal-derived hepatic organoids displayed higher expansion capacity than adult donor-derived human organoids [33,44]. We examined different conditions of EM and identified conditions that were associated with the expansion of organoids and upregulation of gluconeogenic targets. Our data suggest that the supplemented molecules may act in a concerted manner in order to expand organoids, increase HNF4α, which regulates hepatocyte maturation [69] and relatively increase gluconeogenic hepatocyte expression, or in an opposing manner to limit these effects. Although these findings shed some light on the roles that these growth factors and small molecules play in expansion rates, proliferative capabilities, HNF4α, G6PC, PCK1, and ALB expression, more in-depth studies, and molecular analyses of the signaling pathways, are required to assess the full breadth and specific roles of each of these growth factors and signaling molecules in hepatic organoid differentiation and hepatocyte expansion.

We utilized HepG2 cells in serum-free and BSA-free normoglycemic conditions to study the gluconeogenic potential of HepGOs in 3D cultures. HepG2 cells in classic cultures had limited potential to detect hepatotoxicity due to their reduced CYP activity [70]. While CYP activity and detoxification are critical functions of primary hepatocytes, differences between hepatocyte donors necessitate the screening of multiple primary hepatocyte batches [71] for proper assessment of these functions in hepatic organoids. Moreover, Alpha-1-antitrypsin and Ornithine transcarbamylase functions should also be assessed to evaluate the potential use of our hepatic organoids as models to study the pathologies of α1-antitrypsin deficiency and Alagille syndrome patients.

A variety of cells that could contribute to functional liver regeneration have been identified in the fetal and adult livers, both under normal and injury conditions [72]. Mature hepatocytes have been demonstrated to display bi-lineage differentiation potential in vivo by transdifferentiating into biliary epithelial cells [73,74]. However, long-term 3D culture of liver progenitor cells remains challenging [75]. Evidence suggests that liver progenitor cells are not readily detectable based on the expression of adult progenitor cell markers such as Lgr5 in the healthy adult liver [42], but become activated upon injury and could contribute to the remarkable regenerative liver response. A cocktail of three small molecules Y-27632, A8301, and CHIR99021 could convert rat and mouse hepatocytes in vitro into small proliferative bipotent progenitor cells [32]. These cells were reported not to resemble hepatocytes morphologically, yet they retain their proliferative capacity and hepatic differentiation ability [32]. Bipotent epithelial liver organoids could be isolated and expanded from perinatal cells and/or bile ductal fragments and differentiated into mature and functional hepatocytes [34,76]. Downregulation of TGF-β and Notch are highly correlated with the specification of hepatoblast towards the hepatocyte fate [48]. We examined the effects of the TGFβ inhibitor; A8301, which is more potent, in inhibiting TGFβ type I receptor ALK5 (and closely related ALK4/7), than other TGFβ inhibitor analogs, and also prevents phosphorylation of Smad2/3 and the growth inhibition induced by TGFβ [77]. A8301; a reported component of the prior hepatic organoid conditions (albeit utilized at a range of conc. from 50 nM to 4 μM [31,34,42,43]) was shown to extend the time and colony-forming efficiency before cultures eventually deteriorated. A8301, while is an established TGF-β Inhibitor, is also recognized for its Wnt-3α modulatory role and its potential to aid in the maintenance of bipotent hepatoblasts [41,43,78]. A8301 was shown to have little or no effect on BMP type I receptors, p38 mitogen-activated protein kinase, or extracellular regulated kinase [77]. A8301, along with other ALK inhibitors, including LY2157299, SB431542, have been identified to affect stem cell differentiation [79]. We demonstrated that A8301, when used within the reported conc. range, could negatively interact with other factors in the 3D culture media to affect expansion rates and render the gluconeogenic potential with G6PC and PCK1 expression differing upon differentiation. Thus, additional studies are required to examine the specific roles of these receptors in hepatocyte regeneration and function, ideally, by interrogating our single cell-derived organoid models using genome editing and single-cell RNA sequencing assays [80] during the different phases of hepatic organoid development.

OSM, a member of the IL-6 cytokine family, has been linked with the primary formation of the liver bud during embryogenesis [45,46]. OSM signaling is required for hepatocyte proliferation and tissue remodeling during liver regeneration [45,46]. Moreover, OSM was recently found to contribute to glucose homeostasis and insulin resistance [81]. We supplemented two of our organoid expansion conditions with OSM, which allowed; when combined with FSK, expanded organoids to maintain detectable gluconeogenic expression in a spatiotemporal distribution. FSK, a diterpene, is highly efficient at increasing concentrations of cAMP via the activation of adenylate cyclase (AC) [47]. While reported protocols for rapidly expanding liver organoids utilized FSK [34], and FSK was shown to induce proliferation of biliary duct cells in vivo [82], it is unclear if FSK’s effects in adult hepatic organoids are by directly interacting with the catalytic subunit of AC [47,83]. Notwithstanding, FSK appears to be an important compound in the rapid expansion of liver organoids and could have an impact on hepatocyte glucose production and insulin resistance [84]. Collectively, we identified optimized conditions by limiting A8301 and incorporating FSK and OSM to allow the expansion of adult hepatic organoids with gluconeogenic competence.

Gluconeogenesis is the de novo production of glucose from endogenous carbon sources. Enhanced hepatic glucose production causes fasting hyperglycemia in T2DM patients. T2DM is also an emerging risk factor for NASH progression to advanced fibrosis, cirrhosis, and HCC [3]. A consensus panel has proposed to rename NAFLD a metabolic-dysfunction-associated fatty liver disease (MAFLD) based on the presence of obesity, T2DM, and hepatic metabolic dysregulation [85]. Primary hepatocytes are the current gold standard for in vitro metabolic dysregulation studies of gluconeogenesis. Similar to primary hepatocytes, hepatoma cells have been expanded in 3D cultures as 3D spheroid models for use in drug metabolism and liver function assays [35,36]. Previous studies suggested that glucose release is mainly compensated for by glycogenolysis in the basal state but is provided by gluconeogenesis in the presence of glucose substrates [86]. Moreover, HepG2 cells in serum-containing spheroid culture demonstrated less glucose metabolism abilities in terms of glucose consumption, intracellular glycogen content, gluconeogenesis rate, and sensitivity to glucose modulator hormones such as glucagon [87]. We demonstrated that HepGOs in serum-free and BSA-free defined 3D organoid culture contain cells with hepatocyte-like function and metabolic competence responding to glycerol and hormonal glucagon stimuli, suggesting that HepGOs could be further developed for glucose metabolism studies. We have examined multiple media conditions for generating hepatic organoids and assessed their ability to generate glucose and utilize different substrates for gluconeogenesis. We provide increased knowledge on the optimum conditions for the generation of hepatic organoids to support future incorporation of these platforms in clinical settings.

## Supplementary Materials

The following are available online at https://www.mdpi.com/article/10.3390/cells10123280/s1, Figure S1: Different expansion media (EM) conditions and examples of hepatic organoids, Figure S2: Differentiation of hepatic organoids, Figure S3: Colocalization of markers for volumetric analysis of 3D cultured organoids, Table S1: Overview of the conditions used for generation of Wnt-3α and R-Spondin-1 factors, Table S2: Overview of the Expansion Media (EM) conditions used, Table S3: List of primers used, Table S4: List of antibodies and assay conditions.
