# Supplementary material for: Optimized 3D Culture of Hepatic Cells for Liver Organoid Metabolic Assays
Source: Cells. 2021 Nov 24;10(12):3280. doi: 10.3390/cells10123280 (PMC8699701; doi:10.3390/cells10123280)
Supplement: Supplementary file 1 [file cells-10-03280-s001.zip › cells-1436684-supplementary.pdf]

# Optimized 3D culture of hepatic cells for liver organoid metabolic assays

**Christian Moya Gamboa, Yujue Wang, Huiting Xu, Katarzyna Kalemba, Fredric E Wondisford, Hatem E Sabaawy**

**Supplementary Data**

A

| 3D organoid culture EM conditions for adult hepatocytes (HepAOs) | | | | | |
|---|---|---|---|---|---|
| **C1** | **C2** | **C3** | **C4** | **C5** | **C6** |
| EM | EM + FSK + A83-01 | EM + FSK | EM + A83-01 | EM + OSM (0-6d)+ BMP7 (7d+) | EM + FSK + OSM (0-6d)+ BMP7 (7d+) |

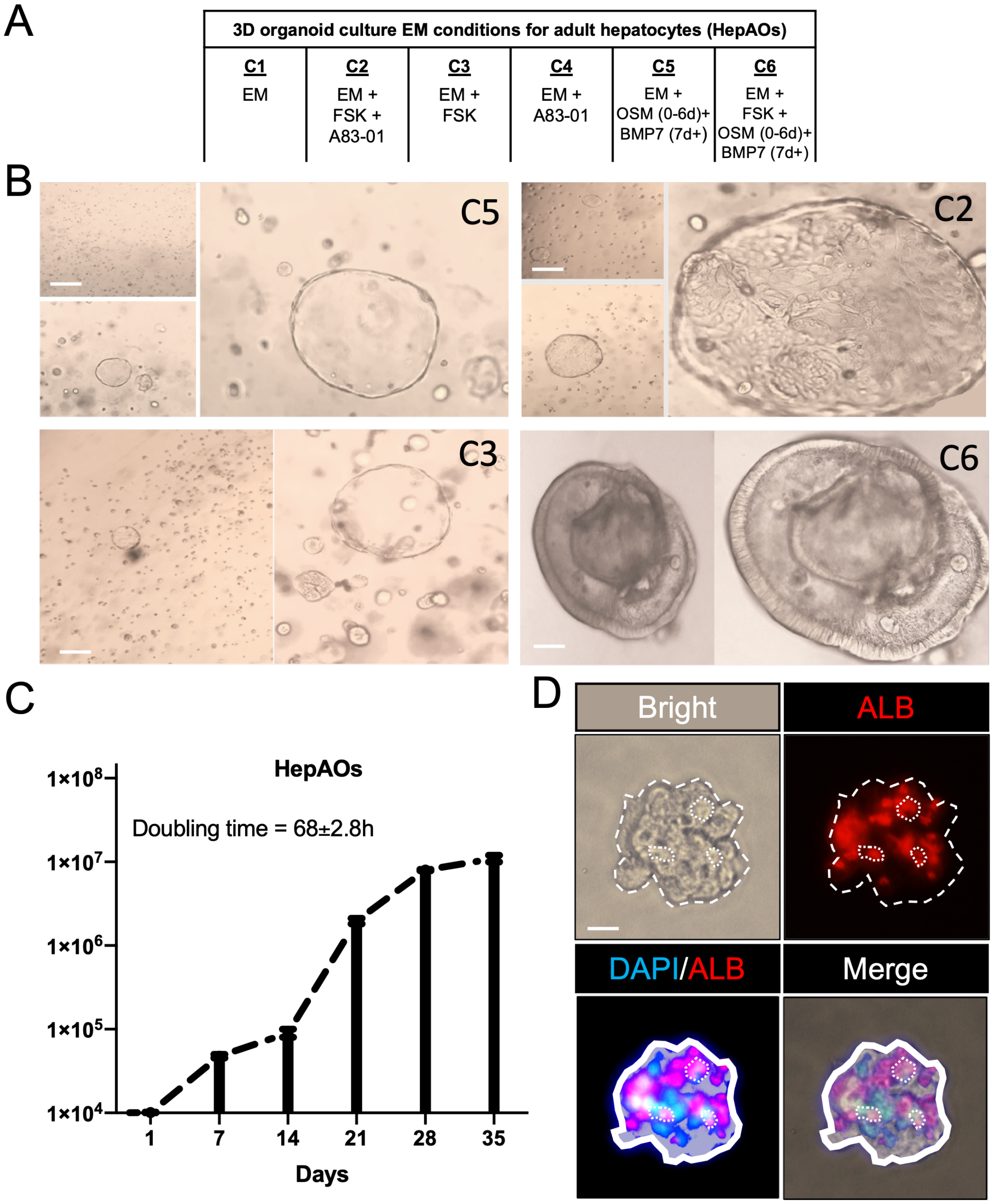


**Figure S1.** Different expansion media (EM) conditions and examples of hepatic organoids. (**A**) List of 3D organoid EM culture conditions examined for expansion of donor derived hepatocytes. (B) Representative images of HepAOs generated in EM supplemented with Forskolin (FSK), A83-01, and Oncostatin M (OSM). Note that not all plated cells expand into organoids, supporting the reported organoid forming potential from ADC and/or organoid forming cells. (**C**) Proliferation rate of single cell derived HepAOs and in vitro growth rate at the indicated time in EM. Doubling time is indicated in day 21-28. (**D**) IF and overlay of ALB (red) expressing cells in the organoids. The typical nuclear DAPI (blue) and cytosolic ALB (red) staining are outlined in overlayed and merged images of 3D organoids. Scale bar is 100 µm.

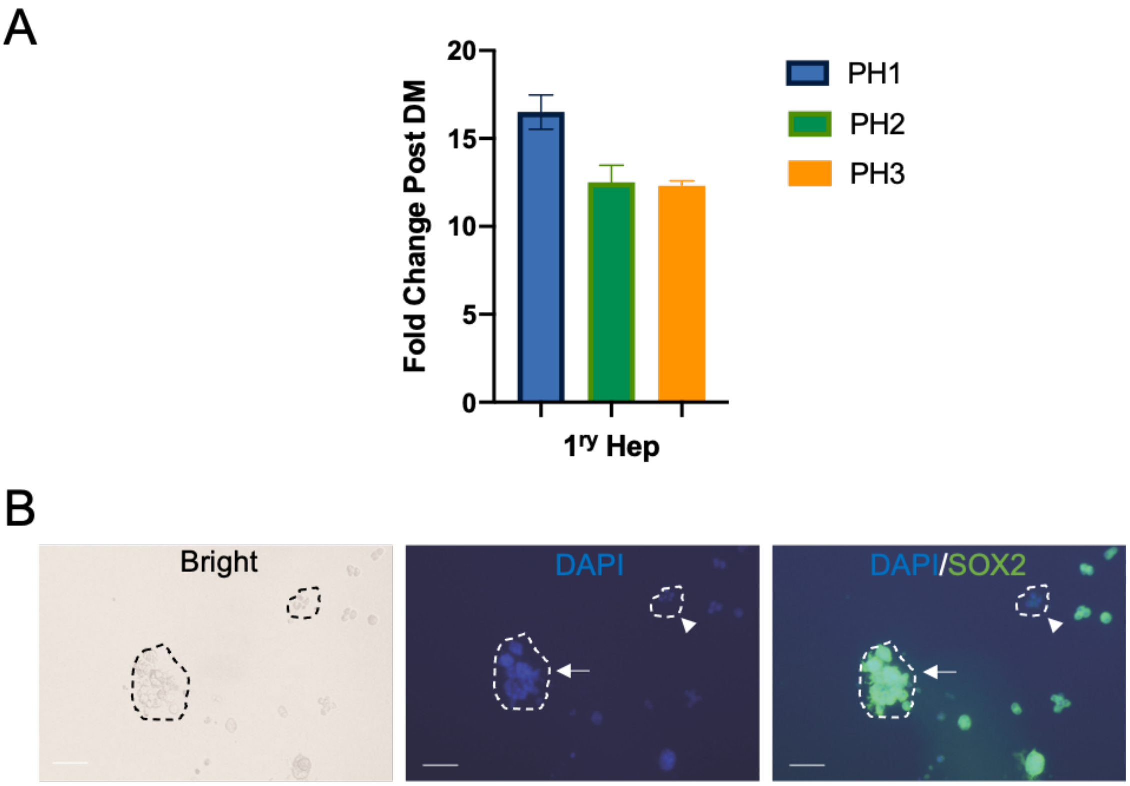


**Figure S2.** Differentiation of hepatic organoids. (**A**) Fold change in the expression of PKC1 post induction of differentiation in differentiation media (DM) in HepAOs from three donors. The difference between donors was not significant. (**B**) Expression of SOX2 in expanded organoids. Note the nuclear staining of SOX2 in organoids (arrow), while smaller cell structures (arrowhead) didn't show staining. DAPI, nuclear marker in blue, SOX2 in green. Scale bar is 100 mm.

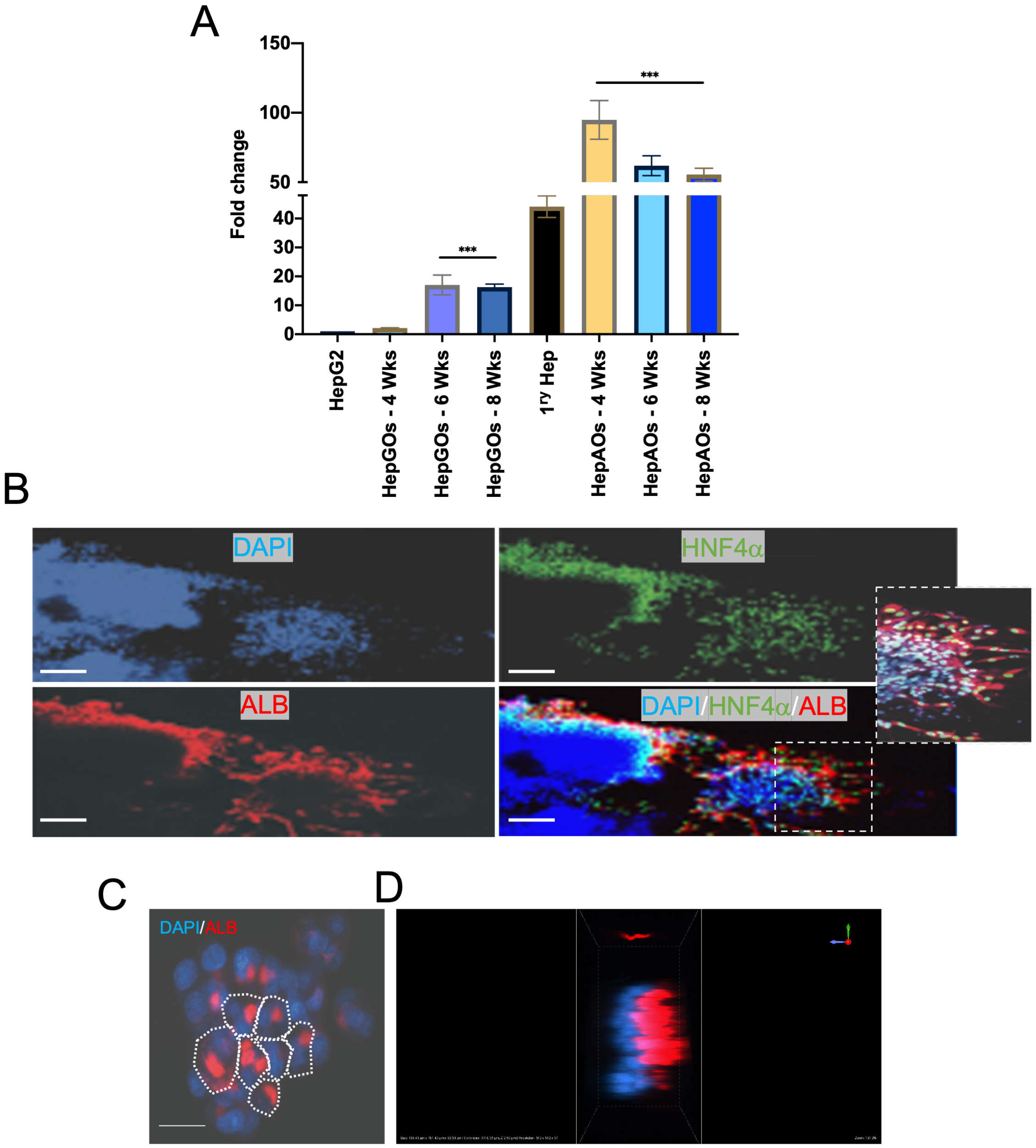


**Figure S3.** Colocalization of markers for volumetric analysis of 3D cultured organoids. (**A**) HNF4α expression in HepGOs and HepAOs compared to HepG2 cells and hepatocytes respectively at the indicated time points. (**B**) Images using DAPI (blue) for nuclear staining and showing the expression of HNF4α, a marker used to determine hepatocyte fates and ALB (red) as a functional hepatocyte marker. ALB expression is mainly observed around the periphery of the large, differentiated organoids. The outlined area is a z-stack confocal image. (**C**) IF images of a representative organoid used for volumetric analysis. The typical nuclear DAPI (blue) and cytosolic ALB (red) staining are outlined. (**D**) Representative volumetric ALB/DAPI overlay used for size estimates of organoids. Note that DAPI (blue) and cytosolic ALB (red) staining are at different planes. This organoid was determined to approximately measure 181.43 ◎m deep X 92.50 ◎m wide. Scale bar is 100 µm. (***p<0.001).

| Media | Components | Storage |
|---|---|---|
| Wnt-3α Growth Media | DMEM supplemented with 10% FBS, 1% Penicillin/ streptomycin, and 2% G418 | 4ºC for one week |
| Wnt-3α Harvest Media | DMEM supplemented with 10% FBS, 1% Penicillin/ streptomycin | 4ºC for one month |
| R-Spondin-1 Growth Media | DMEM supplemented with 10% FBS, 1% Penicillin/ streptomycin, and 200-300ug/mL zeocin | 4ºC for one week |
| R-Spondin-1 Harvest Media | Advanced DMEM/F-12 supplemented with 1% Penicillin and Streptomycin ,1% Glutamax, and 10mM HEPES. | 4ºC for one month |

**Table S1.** Overview of the conditions used for generation of Wnt3α and R-Spondin-1 factors. Media specifications, and storage times are indicated.

| Name | Components | Storage |
|---|---|---|
| **Basal Medium** | Advance DMEM/F-12 supplemented with 1% Penicillin and Streptomycin, 1% Glutamax, and HEPES 10mM. | 4ºC for 1 month |
| **Wash Medium** | DMEM (High Glucose, Glutamax, and Pyruvate) supplemented with 1% FBS, and 1% Penicillin/Streptomycin. | 4ºC for 1 month |
| **Human Liver Expansion Medium (EM)** | Basal Medium supplemented with 1:50 B27 Supplement (without vitamin A), 1:100 N2 Supplement, 1 mM N-acetylcysteine, 10% (v/v) R-spondin1 conditioned medium, 10mM Nicotinamide, 10 nM recombinant human [Leu15]- Gastrin-I, 50 ng/mL recombinant human EGF, 100 ng/mL recombinant human FGF10, and 25 ng/mL recombinant human HGF. | 4ºC for 2 Weeks |
| **Human Liver Isolation Medium** | Liver Expansion Medium supplemented with 25 ng/mL recombinant human Noggin, 30% (v/v) Wnt-3α conditioned medium, and 10 nM Rho Kinase (ROCK) Inhibitor (Y-27632). | 4ºC for 2 Weeks |
| **Human Hepatocyte Differentiation Medium (DM)** | Basal Medium supplemented with 1:50 B27 Supplement (without vitamin A), 1:100 N2 Supplement, 1 mM N-acetylcysteine, 10 nM recombinant human [Leu15]-Gastrin I, 50 ng/mL recombinant human EGF, 0.5 μM A8301, 10 μM DAPT, 3 μM Dexamethasone, 25 ng/mL BMP7, and 100 ng/mL recombinant human FGF19. | 4ºC for 2 Weeks |

**Table S2.** Overview of the expansion media (EM), Differentiation media (DM) and other media conditions used for 3D hepatic organoid culture.

| Target | Forward Sequence (5' - 3') | Reverse Sequence (5' - 3') |
|---|---|---|
| **B-Actin** | GAC CTG ACT GAC CTC AT | TCT CCT TAA TGT CAC GCA CG |
| **G6PC** | ACG TGA TGG TCA CAT CTA CTCT | ACA TTC AAG CAC CGA AAT CTG |
| **PCK1** | ACG TAC ATG GTG CGA CCT TT | ATC CCC AAA ACA GGC CTC AG |
| **HNF4a** | GCA TCT TCT TTT GCG TCG | TGT AAA CCA TGT AGT TGA GGT |

**Table S3.** List of SYBR green Q-PCR primers used. Description of the primers used for hepatocyte specific expression.

| Manufacturer | AB Type | Target | Species | Dilution | Reference |
|---|---|---|---|---|---|
| **Bethyl Labs** | Primary | Albumin | Goat | 1:200 | A80-229A |
| **Invitrogen** | Primary | HNF4α | Mouse | 1:100 | 417700 |
| | DAPI | Nucleic Stain | | 1:200 | |
| **Invitrogen** | Secondary | Goat | Donkey | 2µg/ml | A11057 |
| **Invitrogen** | Secondary | Mouse | Donkey | 1:2000 | A21202 |

**Table S4.** List of antibodies used. Description of the antibodies used for IF assays on organoids.
